# Fundamentality from grounding trees

**DOI:** 10.1007/s11229-021-03054-2

**Published:** 2021-03-13

**Authors:** Fabrice Correia

**Affiliations:** Department of Philosophy, University of Geneva, Rue De-Candolle 5, 1211 Geneva 4, Switzerland

**Keywords:** Fundamentality, Relative fundamentality, Absolute fundamentality, Grounding, Immediate grounding

## Abstract

I provide and defend two natural accounts of (both relative and absolute) fundamentality for facts that do justice to the idea that the “degree of fundamentality” enjoyed by a fact is a matter of how far, from a ground-theoretic perspective, the fact is from the ungrounded facts.

What is it for a fact to be more fundamental than another fact? There are plausibly numerous answers to this question, pointing to different relations of *being more fundamental than*. In this paper, I wish to explore the idea that *being more fundamental than* is a matter of how far, ground-theoretically speaking, the facts that it relates are from the ungrounded facts. My main aim is to make this idea precise.

The idea has certainly been contemplated by many, but it is only very recently that philosophers have attempted to discuss it in detail. Karen Bennett (2017) initiated the move, and one can indeed extract from her discussion an account of *being more fundamental than* understood in terms of ground-theoretic distance from the ungrounded facts (an account which she does not endorse, see below). This account is flawed in a number of ways, as Bennett herself admits. In what follows, I offer two natural accounts in the same spirit, each with its own merits, which are better than Bennett’s account. These two accounts correspond to two ways of measuring distances from the ungrounded, “from the top down” and “from the bottom up” which, perhaps surprisingly, do not always give the same results. I also discuss a similar account recently put forward by Jonas Werner (forthcoming). What his account precisely amounts to is not clear, but I propose a reconstruction of the account that strikes me as being as close as one could wish to Werner’s intentions. My version of Werner’s account turns out to be extensionally equivalent to my bottom-up account. I also show that a top-down Werner-style account can be provided, and that it is extensionally equivalent to my own top-down account. Despite these facts of extensional equivalence, I argue that my accounts are superior to their Werner-style counterparts.

The plan of the paper is as follows. In Sect. 1, I spell out the Bennettian account of *being more fundamental than* and explain why it is inadequate. In Sect. 2, I introduce my top-down account and in Sect. 3 my bottom-up account. In Sect. 4, I spell out the Werner-style accounts and argue that mine are to be preferred. In Sect. 5, I close the discussion by providing accounts of the relation of *being as fundamental as* and accounts of the property of *being fundamental* that mesh well with my accounts of *being more fundamental than*. In the “Appendix”, some important claims made in previous sections are established.^1^^,^^2^

## Bennett

Bennett (2017) identifies a class of “building relations”, which comprises composition, constitution, set-formation, realisation, microbased determination and grounding (pp. 8–13), and proposes a definition of *being more fundamental than* indexed to a building relation R that goes as follows (p. 161):

Entity x *is more fundamental*_*R*_* than* entity y iff_df_ at least one of the following holds:

1. x is fewer R-ing steps away from the non-R-ed entity(ies) that terminate its unique chain than y is from the non-R-ed entity(ies) that terminate its unique chain;
2. x at least partially R-s y;
3. x stands in the ancestral of partial R-ing to y;
4. x is not R-ed whereas y is R-ed;
5. x belongs to some kind K and y belongs to some kind K* such that
  - neither K nor K* includes both R-ed and non-R-ed members, and
  - y does not belong to K and x does not belong to K*, and
  - K*s are typically or normally R-ed in Ks.

Irrespective of the underlying building relation, the highly disjunctive nature of the *definiens* suggests that Bennett merges distinct notions into one, and it is legitimate to wonder whether the resulting notion is natural enough to be worthy of interest. I will leave this issue aside here and focus only on clause (1) of the definition in the particular case where R is grounding.^3^ This instance of clause (1) characterises a notion of *being more fundamental than* that fits well with the conception of fundamentality I am interested in here. Let me label this notion ‘*being more Bennett-fundamental than*’, and accordingly adopt the following definition:

Fact F *is more Bennett-fundamental than* fact G iff_df_ F is fewer grounding steps away from the ungrounded fact(s) that terminate its unique chain than G is from the ungrounded fact(s) that terminate its unique chain.

**Fig. 1:**
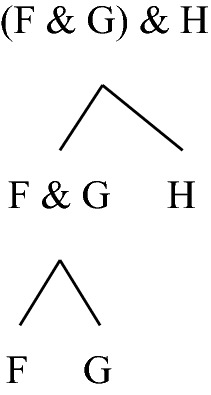
A tree for a conjunctive fact

There are obvious problems with this definition, which Bennett herself mentions (but in the more general context of a search for a definition of *being more fundamental than* indexed to an arbitrary building relation; here and below I pretend that she had only grounding in mind). One problem is that since grounding is sometimes many-one rather than one–one,^4^ it sometimes generates (proper) *trees* rather than chains. Conjunctive facts provide straightforward illustrations. Granted that (F & G) & H is grounded in F & G and H taken together, and that F & G is grounded in F and G taken together, the structure that witnesses how (F & G) & H “arises from” F, G and H is something like the one depicted in Fig. 1.

**Fig. 2:**
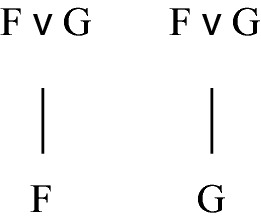
Two trees for a disjunctive fact

Another problem is that such grounding trees or chains need not have a finite height. Many examples of grounding trees or chains that never “bottom out” can be found in the literature (see e.g. Correia 2005: pp. 63–64 and Rosen 2010: p. 116 for early references). A third problem is that some facts may be associated with more than one grounding tree or chain. Disjunctive facts illustrate this. Granted that F ∨ G is grounded in F and also in G, we have two associated structures rather than one, as shown in Fig. 2.

There is a further problem with the definition, which Bennett does not highlight. It relies on the presupposition that grounding operates by discrete steps. The *definiens* is indeed best understood as invoking the notion of *immediate* grounding: the chains Bennett has in mind are chains in which each fact that is not last is immediately grounded in the next fact in the chain. Let us take such a notion for granted. Then there arises the question of whether there can be facts that are grounded but which have no immediate grounds—*transcendent facts*, as I will call them. (I assume that the question of whether there can be facts that are immediately grounded but not grounded does not arise: *being immediately grounded* surely entails *being grounded*.) It is far from absurd to hold that there are such facts. Thus, consider a body B whose mass is 1 kg, and consider the fact F_1_ that B has a mass that is between 0 and 2 kg. We may take F_1_ to be grounded, say in the fact that B has a mass that is between 0.5 kg and 1.5 kg, and also in the fact that B has a mass that is between 0.75 kg and 1.25 kg, and so on. Yet there does not appear to be any fact that *immediately* grounds F_1_.^5^ Now suppose there *are* transcendent facts. Since they are not immediately grounded, they are *zero* grounding steps away from any facts, and therefore they are more Bennett-fundamental than any facts that have immediate grounds. Does this not make *being more Bennett-fundamental than* ill-suited to capture a *bona fide* notion of relative fundamentality of the sort I am trying to capture? I am not suggesting that the answer is positive. The problem that I want to highlight here is that the definition should come with a story about what to do with transcendent facts, and that this story remains to be told.

The definition of *being more Bennett-fundamental than* is thus problematic in several respects. However, as I show in the next section, it is possible to formulate a definition of *being more fundamental than* that preserves the spirit of the Bennettian definition but which fails to have the shortcomings that have just been highlighted.

## Defining being more fundamental than by measuring distances “from the top down”

The definition is framed in terms of trees, more precisely in terms of trees as they are defined in set theory.^6^ A set-theoretic (rooted) *tree* is a structure 〈T, <〉 that satisfies the following conditions:

- T (the set of *nodes*) is a non-empty set;
-  < (*precedence*) is a strict partial order on T—i.e. an irreflexive and transitive binary relation on T;
- For any x ∈ T, the set {y ∈ T: y < x} of predecessors of x is well-ordered by < —i.e. < is a total order on the set, and any of its non-empty subsets has a minimal element for < (which must be unique since < is total on the set in question);
- T has a unique minimal element for < (the *root* of the tree).

A *leaf* in a tree is a node that has no successor for the partial order. A *parent* is a node that is not a leaf. A *child* of a node x is a node that immediately follows x, i.e. a node y such that x precedes y and there is no node z such that x precedes z and z precedes y. A *branch* is a set of nodes totally ordered by precedence that is not strictly contained in another such set of nodes.

**Fig. 3:**
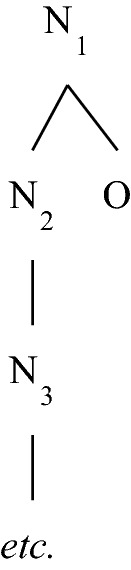
Another tree (a bush with an infinite branch)

Trees are typically represented “upside down” in diagrams such as those of the previous section. The diagram in Fig. 3 is a further illustration. It represents a tree with nodes N_1_, N_2_, N_3_, … and O, such that a node precedes another node iff there is a path from the former to the latter that goes exclusively downward. The root of the represented tree is N_1_. This tree has only one leaf, O, and its parent nodes are thus N_1_, N_2_, N_3_, …. And it has two branches, the set {N_1_, N_2_, N_3_, …} and the set {N_1_, O}.

The trees needed to formulate the account are special kinds of trees, “small” trees that I call *bushes*. The definition of a bush goes just like the definition of a tree given above except that the third condition is replaced by the stronger condition that for any node x, the set of predecessors of x is totally ordered by the precedence relation *and finite*.

**Fig. 4:**
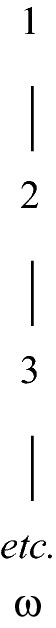
A tree that is not a bush

In any tree, be it a bush or not, any node that has successors (i.e. any parent) must have immediate successors (i.e. children). The dual property is possessed by all bushes, but not by all trees: in any bush, but not in any tree, any node that has predecessors (i.e. any node distinct from the root) must have an immediate predecessor (i.e. must be a child). An example of a tree that fails to have that property, and hence is not a bush, is 〈{1, 2, 3, …, ω}, < 〉 where < is the usual ordering on the ordinals. It is represented in Fig. 4.

**Fig. 5:**
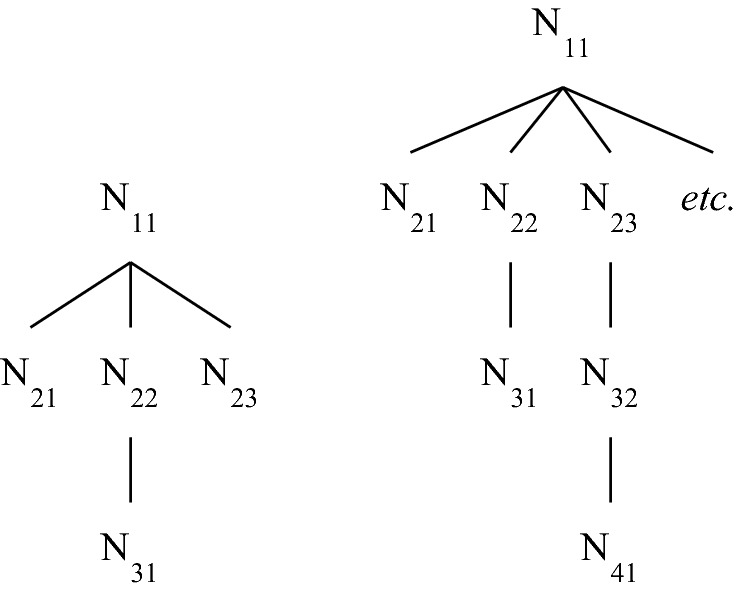
Two bushes with finite branches

The tree represented in Fig. 3 is a bush that has an infinite branch. Figure 5 represents two bushes without infinite branches—the first one with a finite number of branches, the second one with infinitely many branches.

Every tree can be assigned an ordinal number usually called its *height*, which provides a measure of its “vertical length”. I will call this ordinal number a *T-height* (‘T’ is for ‘Top’), to distinguish it from another kind of tree-theoretic height that I will define in the next section. Define first the *height of a node* x in a tree as the ordinal that is the order-type of (i.e. is order-isomorphic to) the set of x’s predecessors. The *T-height of a tree* is then defined as the least upper bound of the set {height of (x) + 1: x a node in the tree}.

In case the tree is a bush, the height of a node is a finite ordinal, it is simply the number of its predecessors in the tree. The T-height of a bush is either a finite ordinal or ω. The smallest T-height for a bush is 1, this is the T-height of any bush with only one node. The first bush depicted in Fig. 5 has T-height 3, and the second one T-height ω. The bush depicted in Fig. 3 also has T-height ω, but for a different reason: it has T-height ω because it contains an infinite branch, whereas the second bush depicted in Fig. 5 has T-height ω because it contains branches of arbitrarily big finite cardinality. (By the general definition of T-height, the tree depicted in Fig. 4—which, again, is not a bush—has T-height ω + 1.)

The last mathematical notion needed before proceeding with our main concern is that of a *labelled* tree: a labelled tree is a tree together with a function that assigns to each node of the tree an entity, which is then said to *label* or to *occupy* the node.

The notions introduced so far have nothing to do with grounding. Let me now lay down the following ground-theoretic definitions:

- A *grounding tree* is a bush labelled by facts (i.e. whose nodes are occupied by facts), which satisfies the condition that every fact occupying a parent node (if any) is immediately grounded in the facts that occupy the corresponding children. Each grounding tree thus represents a ground-theoretic “genealogy” of the fact that occupies its root; it represents in a neat way how links of immediate grounding are chained together up to this fact. Note that grounding trees can be used to provide a straightforward definition of mediate grounding: fact F is *mediately* grounded in the members of set of facts Γ iff_df_ there is a grounding tree without infinite branches whose root is occupied by F and whose leaves are the members of Γ.
- A grounding tree *for a fact* is a grounding tree whose root is occupied by that fact.
- A *complete* grounding tree is a grounding tree whose leaves (if any) are occupied by facts that fail to be immediately grounded. A complete grounding tree for a fact thus represents a complete genealogy of that fact in terms of immediate grounding, a genealogy that goes as far as possible in the grounded-to-grounding direction. Note that grounding trees that have no leaves, i.e. that have only infinite branches, count as complete.
- Where $\mathcal{J}$ is a grounding tree for fact F, F’s *T-height* in $\mathcal{J}$ is $\mathcal{J}$’s T-height.
- The *T-rank* of a fact is the smallest of its T-heights.

If a fact fails to be immediately grounded, then there are complete grounding trees for that fact, and all of them consist of one node occupied by this fact. Facts that fail to be immediately grounded therefore have T-rank 1. It can be proved that for every fact that is immediately grounded, there are complete grounding trees for that fact (see Proposition 15 in the “Appendix”). This guarantees that every fact has a T-rank. Facts that are immediately grounded have T-ranks that are comprised between 2 and ω.

My suggestion for defining *being more fundamental than* goes as follows:

(T-MFT): F *is more fundamental*_*T*_* than* G iff_df_ F’s T-rank is smaller than G’s T-rank.

As good as the account might seem to be, however, it cannot be accepted without further comments. At the end of the previous section, I emphasised that one cannot lightly rule out that some facts are transcendent, i.e. grounded without being immediately grounded. And I also argued that, accordingly, the definition of *being more Bennett-fundamental than* has to be supplemented with a story about what to do with transcendent facts. The same is true of (T-MFT). Transcendent facts have T-rank 1. Consider two distinct ungrounded facts F_0_ and G_0_ and their conjunction F_0_ & G_0_. Granted that F_0_ & G_0_ is immediately grounded in F_0_ and G_0_ taken together, and that it is grounded in nothing else, it has T-rank 2. Given (T-MFT), it follows that all transcendent facts are more fundamental than F_0_ & G_0_. Is this an acceptable consequence?

I do not have firm “intuitions” about the view that all transcendent facts are more fundamental than F_0_ & G_0_, and more generally about the view that all transcendent facts are more fundamental than all facts that have immediate grounds. Sometimes I am tempted to say that the general view is perfectly acceptable: on the conception of fundamentality that I am exploring here, it is the relation of immediate grounding that generates the relation of *being more fundamental than*, and since transcendent facts are not immediately grounded, they should count as being minimal for that relation. But I am also sometimes tempted to say that the general view is not acceptable. For lack of proper arguments in favour or against the view, I officially offer (T-MFT) not as an account that is acceptable *simpliciter*, but as an account that is acceptable *on the assumption that there are no transcendent facts*, and I officially remain agnostic on the question of whether it is acceptable *simpliciter*. Of course, I take it that this move is also open to friends (actual or merely possible) of the Bennettian account.

The proposed account of *being more fundamental than* is clearly in the spirit of the Bennettian account discussed in the previous section. Remember, the target idea is that relative fundamentality is a matter of how far, from a ground-theoretic point of view, the facts are from the ungrounded facts. Given the assumption that there are no transcendent facts, the ungrounded facts are the facts that are not immediately grounded. On that account, the target idea is respected to the letter if we focus on facts of finite T-rank; and if we focus on all facts, be they of finite T-rank or not, the original idea is still respected, if not in letter, at least in spirit, since it is certainly acceptable to say that facts of T-rank ω are infinitely far from the ungrounded facts. And it is also clear that my objections to the Bennettian account do not affect my account: the latter respects the fact that grounding is sometimes many-one, the fact that chaining links of immediate grounding may in some cases fail to yield a structure that “starts” with ungrounded facts, the fact that a given fact may be immediately grounded in different ways, and, finally, it comes with a story about what to do with the view that there are transcendent facts.

Before moving on to the next section, let me point to an important feature of the account, and reply to two questions that some may have about the particular way in which I defined grounding trees.

(1) Given (T-MFT), one to easily construct counterexamples to the following principle that many, including Bennett, accept^7^:

If F grounds, or even only helps to ground, G, then F is more fundamental than G.

For consider two facts F and G with, say, F of T-rank 1 and G of T-rank greater than 2. Granted that F immediately grounds F ∨ G, F ∨ G has T-rank 2, the smallest T-rank for a fact that is immediately grounded. And granted that G immediately grounds F ∨ G, G grounds F ∨ G. Yet since G’s T-rank is greater than 2, G is *not* more fundamental than F ∨ G according to (T-MFT).

**Fig. 6:**
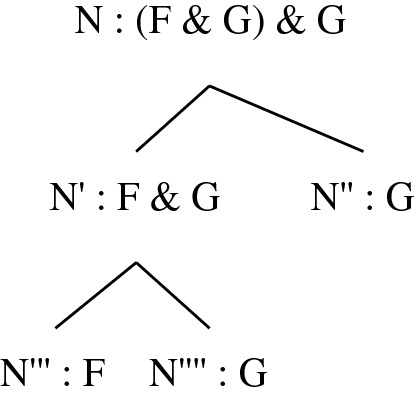
Why using* labelled* trees is important

(2) Why define grounding trees as rooted trees labelled by facts rather than as unlabelled rooted trees whose nodes are themselves facts? The reason, quickly put, is that the same fact can appear twice in a ground-theoretic genealogy of a fact. Consider for instance the labelled tree depicted in Fig. 6. Here the nodes are N, N', N'', N''' and N'''', and the labels are the facts (F & G) & G, F & G, F and G. This is a grounding tree in the sense defined above. G occupies two nodes in this tree. It is clear that no rooted tree whose nodes are among the facts just listed can represent this genealogy. (Note that although the diagrams in Figs. 1 and 2 can be seen as depicting trees whose nodes are the facts that are mentioned, on the assumption that these facts are all distinct, they can alternatively be seen as depicting grounding trees whose labels are the facts in question and whose nodes are represented by the endpoints of the line segments.)

(3) Why define grounding trees in terms of bushes rather than trees in general? The general idea of using trees labelled by facts is this: the relation of parent to children in such trees represents the link between a fact and some facts that immediately ground it. Now consider for instance the tree depicted in Fig. 4 and suppose given a labelling of that tree by means of facts. (The considerations that follow are intended to generalise to all trees that are not bushes.) Following the general idea just mentioned, we can interpret part of the tree in ground-theoretic terms: the fact that occupies k is immediately grounded in the fact that occupies k + 1 for all positive integers k. But what are we going to say about the fact that occupies ω? There are several conventions that we may decide to adopt. For instance, we may agree that in a labelled tree based on the tree in question, the fact that occupies ω is represented as immediately grounding the fact that occupies 1; or that it is represented as immediately grounding all the facts that occupy a positive integer; or again, that it is represented as being immediately grounded in the fact that occupies 1. If any one of these suggestions is adopted, though, it is clear that appeal to the tree depicted in Fig. 4 is dispensable: the corresponding connections can be just as well represented using bushes. More generally, any sensible interpretation of labelled trees based on this tree will make dispensable appeal to such a tree, for the same reason.

## Defining being more fundamental than by measuring distances “from the bottom up”

Complete grounding trees allow one to define a notion of rank that is distinct from that of T-rank and accordingly to provide an alternative definition of *being more fundamental than* on the model of (T-MFT). Interestingly, the rank of a fact in this new sense—its *B-rank*, as I will call it—is finite iff its T-rank is finite, and when finite, the B-rank and the T-rank of a fact are the same; yet when they are infinite, they sometimes differ.

Let me start by adopting the following definitions:

- A *grounding tie* is a pair 〈Γ, F〉, where Γ—the *tail*—is a set of facts and F—the *head*—a fact such that F is immediately grounded in the members of Γ.
- A grounding tie 〈Γ, F〉 is *implemented* at a node x in a grounding tree iff x is a parent node that is occupied by F and the members of Γ are the facts that occupy x’s children.
- A *path* is a nonempty sequence of grounding ties indexed by an interval of integers, which satisfies the condition that for all grounding ties 〈Γ_1_, F_1_〉 and 〈Γ_2_, F_2_〉 such that 〈Γ_1_, F_1_〉 immediately precedes 〈Γ_2_, F_2_〉 in the sequence, F_1_ ∈ Γ_2_.^8^ Note that this condition is trivially satisfied by sequences consisting of just one grounding tie. If a path has a last element, the path is said to be *to* the head of this element.
- A grounding tree *generates* a path iff the elements of the path are all implemented at nodes of the tree.

Let $\mathcal{J}$ be a grounding tree for a fact F that does not generate infinite paths (the condition is crucial).^9^ Where G is a fact in $\mathcal{J}$, let IG(G, $\mathcal{J}$) (‘IG’ is mnemonic for ‘immediate grounds’) be the set of all facts H such that, in $\mathcal{J}$, G occupies a node that is a parent of a node occupied by H (so that $\mathcal{J}$ witnesses the fact that G is immediately grounded in H or in H together with other facts). For every nonnull ordinal α, I define the set of facts BH(α, $\mathcal{J}$) (‘BH’ is mnemonic for ‘B-height’) by transfinite induction as follows:

- BH(1, $\mathcal{J}$) is the set of all facts G in $\mathcal{J}$ such that IG(G, $\mathcal{J}$) = ∅;
- BH(α + 1, $\mathcal{J}$) is the set of all facts G in $\mathcal{J}$ such that (i) IG(G, $\mathcal{J}$) ⊆  ∪ _1 ≤ β ≤ α_ BH(β, $\mathcal{J}$) and (ii) IG(G, $\mathcal{J}$) ∩ BH(α, $\mathcal{J}$) ≠ ∅;
- For α a nonnull limit ordinal, BH(α, $\mathcal{J}$) is the set of all facts G in $\mathcal{J}$ such that (i) IG(G, $\mathcal{J}$) ⊆  ∪ _1 ≤ β < α_ BH(β, $\mathcal{J}$) and (ii) for all nonnull α* < α, there is some β such that α* ≤ β < α and IG(G, $\mathcal{J}$) ∩ BH(β, $\mathcal{J}$) ≠ ∅.

(The definition can be simplified by only keeping the last clause and take it to hold for all ordinals, but its present formulation is more intuitive.) By construction, every fact in $\mathcal{J}$ belongs exactly to one BH(α, $\mathcal{J}$) (see Proposition 1 in the “Appendix”; to prove that every fact belongs to at least one BH(α, $\mathcal{J}$), one needs to use the assumption that $\mathcal{J}$ does not generate infinite paths—hence the requirement that $\mathcal{J}$ has that property). Note that for any nonnull ordinal α, if F (the fact that occupies $\mathcal{J}$’s root) belongs to BH(α, $\mathcal{J}$), then (i) no other fact in $\mathcal{J}$ does and (ii) BH(β, $\mathcal{J}$) = ∅ for all β > α.

**Fig. 7:**
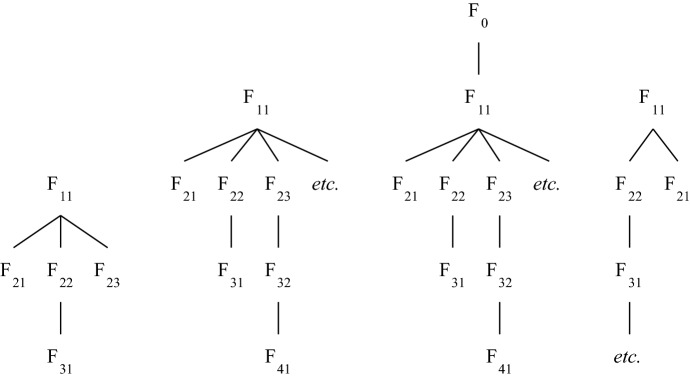
Function BH on various trees

Function BH(…, $\mathcal{J}$) provides a measure of the distance that separates arbitrary facts in $\mathcal{J}$ from the facts that occupy the leaves in $\mathcal{J}$. To illustrate how it does this, consider the first three complete grounding trees (starting from the left) represented in Fig. 7 (the fourth tree has an infinite branch and therefore BH is not defined on it), and call them ‘$\mathcal{J}$_1_’, ‘$\mathcal{J}$_2_’ and ‘$\mathcal{J}$_3_’, respectively. Assume first that the facts mentioned are all distinct. Then:

- BH(1, $\mathcal{J}$_1_) = {F_21_, F_31_, F_23_}, BH(2, $\mathcal{J}$_1_) = {F_22_} and BH(3, $\mathcal{J}$_1_) = {F_11_}.
- For all finite ordinals α ≥ 1, BH(α, $\mathcal{J}$_2_) = {F_βα_: β a finite ordinal with β ≥ 2} and BH(ω, $\mathcal{J}$_2_) = {F_11_}.
- BH(α, $\mathcal{J}$_3_) = BH(α, $\mathcal{J}$_2_) for all α ≤ ω, but we have BH(ω + 1, $\mathcal{J}$_3_) = {F_0_}.

Relaxing the assumption that the facts mentioned in the trees are all distinct may induce changes. Thus, for instance, if in $\mathcal{J}$_2_ F_22_ is identical with F_23_ but not with any other fact, then F_22_ belongs to BH(3, $\mathcal{J}$_2_) rather than to BH(2, $\mathcal{J}$_2_).

Function BH being defined, I can proceed to define B-rank:

- The *B-height of a grounding tree* $\mathcal{J}$ that does not generate infinite paths is the ordinal α such that F ∈ BH(α, $\mathcal{J}$), where F is the fact that occupies $\mathcal{J}$’s root.
- Where $\mathcal{J}$ is a complete grounding tree for fact F, F’s *B-height* in $\mathcal{J}$ is $\mathcal{J}$’s B-height.
- A fact is *bounded* iff there are complete grounding trees for that fact that do not generate infinite paths.
- The *B-rank* of a bounded fact is the smallest of its B-heights.

It can be proved that for any nonnull *finite* ordinal α, a fact has B-rank α iff it has T-rank α (this is Theorem 4 in the “Appendix”). When it comes to infinite ranks, things are different: as we saw, while T-ranks cannot mathematically exceed ω, B-ranks can. I will give a concrete example of a fact with B-rank greater than ω at the end of this section.

I can finally formulate my second definition of *being more fundamental than*:

(B-MFT): F *is more fundamental*_*B*_* than* G iff_df_ F’s B-rank is smaller than G’s B-rank.

Since only the bounded facts have a B-rank, the definition identifies a relation between bounded facts: if a fact is unbounded, it cannot be more or less fundamental_B_ than another fact. By contrast, unbounded facts have T-rank ω and can therefore be less (but not more) fundamental_T_ than other facts. For bounded facts we have the following relations between the two notions of *being more fundamental than*:

- If F is of finite T-rank, then the facts that are more fundamental_B_ than F are those that are more fundamental_T_ than F; likewise the facts that are less fundamental_B_ than F are those that are less fundamental_T_ than F.
- If F is of T-rank ω, then the facts that are more fundamental_B_ than F are those that are more fundamental_T_ than F; no fact can be less fundamental_T_ than F, but some facts may be less fundamental_B_ than F.

Should a choice be made between the two notions of *being more fundamental than*? It depends, I contend, on whether one can rule out as conceptually impossible unbounded facts or facts with a B-rank greater than ω. There are four cases to consider:

1. Both can be ruled out.
2. Fact with a B-rank greater than ω can be ruled out, not unbounded facts.
3. Unbounded facts can be ruled out, not facts with a B-rank greater than ω.
4. None can be ruled out.

In case 1, *being more fundamental*_*T*_* than* and *being more fundamental*_*B*_* than* are as a matter of conceptual necessity coextensive, but the notion of *being more fundamental*_*T*_* than* should be preferred because it is simpler. It should of course also be preferred in case 2. In case 3, it is the other definition that should be preferred. In case 4, there are no principled (as opposed to e.g. pragmatic) reasons to prefer one as opposed to the other. I believe we are in case 4.

For an argument in favour of the conceptual possibility of unbounded facts, consider for instance the following version of the “instantiation regress”, where ‘ /’ signifies that what is immediately on the left in the series is immediately grounded in what is immediately on the right^10^:

The fact that object O instantiates_1_ Fness / The fact that O and Fness instantiate_2_ instantiation_1_ / The fact that O, Fness, and instantiation_1_ instantiate_3_ instantiation_2_ / etc*.*

This series corresponds to a complete grounding tree for the first member of the series that has an (indeed, a unique) infinite branch, and given the plausible assumption that there are no complete grounding trees for this fact without infinite branches, the fact is unbounded. I do not think one can rule out as conceptually impossible the obtaining of such a series of facts, and hence that one can take unbounded facts to be conceptually impossible.

**Fig. 8:**
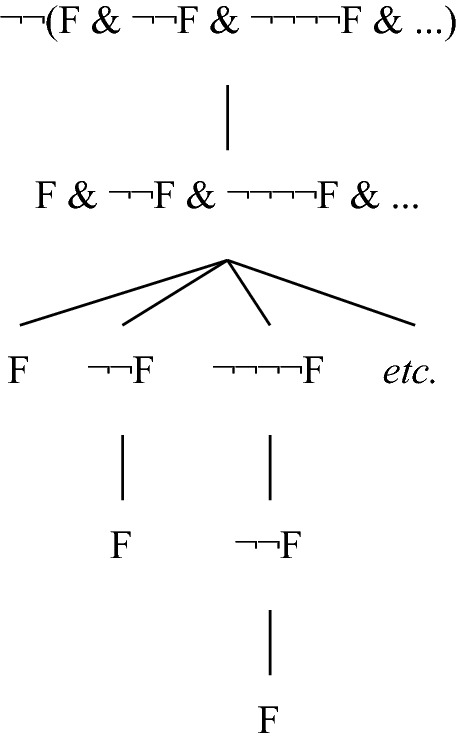
A grounding tree of B-height ω + 1

For the conceptual possibility of facts with a B-rank greater than ω, consider the “concretisation” of the third tree depicted in Fig. 7, depicted in Fig. 8, where it is assumed that F is not immediately grounded (I here take conjunction to be an operation that can take arbitrarily many arguments). Granted that conjunctive facts are immediately grounded in their conjuncts and that for all facts G, ¬¬G is immediately grounded in G, the tree is indeed a complete grounding tree for the fact that occupies its root. The latter fact therefore has B-height ω + 1 in the tree, and consequently its B-rank is greater than ω. I do not think one can rule out as conceptually impossible the view that there are complete grounding trees of the sort under consideration, and hence that one can deem conceptually impossible facts with a B-rank greater than ω.

Thus, I take both (T-MFT) and (B-MFT) to define *bona fide* relations of *being more fundamental than*. Of course, just like I did with (T-MFT), I offer (B-MFT) as an account that is acceptable *on the assumption that there are no transcendent facts*.

## Werner

Werner (forthcoming) puts forward a definition of *being more fundamental than* that is structurally similar to the definitions I proposed in the previous sections: each fact is assigned a rank, which is an ordinal number that is determined by certain structures associated with this fact, and a fact F is said to be more fundamental than a fact G when the rank assigned to F is smaller than the rank assigned to G. One key difference between our respective definitions is that the structures that they invoke are of different kinds. I will suggest that Werner’s ranks are something like my B-ranks. I will also show that something like my T-ranks can be defined in Werner’s framework. As a result, in Werner’s framework just like in mine, two relations of *being more fundamental than* can naturally be defined, one associated with measuring distances “from the bottom up” and the other one with measuring distances “from the top down”. As we will see, it turns out that each Wernerian relation is coextensive the associated relation that I defined in my framework. Despite this result, I will argue that my definitions are to be preferred.

The role played in my framework by complete grounding trees is played in Werner’s framework by what I will call *complete grounding graphs*. Their definition appears last in the following series of definitions (here and below I depart from Werner’s presentation, in a way that will make both the discussion and the comparison with my framework easier):

- A *grounding graph* is a nonempty set of grounding ties.
- A *path in a grounding graph* is a path whose ties are members of the grounding graph.
- A grounding graph $\mathcal{G}$ is said to be *for* fact F iff every member of $\mathcal{G}$ is an element of a path to F in $\mathcal{G}$. Note that since grounding graphs are nonempty, if $\mathcal{G}$ is a grounding graph for F, then F is the head of a grounding tie in $\mathcal{G}$. Also note that whereas a grounding tree must be a grounding tree for a unique fact (the fact that occupies its root), a grounding graph need not be a grounding graph for a fact, and a grounding graph can be a grounding graph for more than one fact.
- If $\mathcal{G}$ is a grounding graph, its *base*, b($\mathcal{G}$), is the set of all the facts that appear in $\mathcal{G}$ but not as heads.
- A grounding graph $\mathcal{G}$ for F is *complete* iff b($\mathcal{G}$) contains only facts that are not immediately grounded.

Consider a given complete grounding graph $\mathcal{G}$ without infinite paths. Werner defines a function ${\text{M}}_{\mathcal{G}}$ that is supposed to assign an ordinal to each fact in $\mathcal{G}$ as follows (I quote the text almost *verbatim*):

- ${\text{M}}_{\mathcal{G}}$ assigns the facts in $\mathcal{G}$ that are not in the head of a grounding tie in $\mathcal{G}$ the ordinal 0.
- If ${\text{M}}_{\mathcal{G}}$ has assigned ordinals to all the facts that immediately partially ground F according to $\mathcal{G}$, then the set of these ordinals O_X_ determines which ordinal is assigned to F by ${\text{M}}_{\mathcal{G}}$ in the following way:
  - If O_X_ has a maximum, then ${\text{M}}_{\mathcal{G}}$ assigns the successor of this maximum to F;
  - If O_X_ doesn’t have a maximum, then ${\text{M}}_{\mathcal{G}}$ assigns the supremum of O_X_ to F.

Then he proceeds essentially (though not literally) as follows: define the *height* of a fact F in a complete grounding graph $\mathcal{G}$ for F without infinite paths as $\text{M}_{\mathcal{G}}\text{(F)}$; then define the *rank* of a fact as the smallest of its heights; and finally define *being more fundamental than* by saying that F is more fundamental than G just in case F’s rank is smaller than G’s rank.^11^

However, Werner’s definition of the ${\text{M}}_{\mathcal{G}}$s is not altogether clear. It somehow looks like an inductive definition, but it does not have the form of such a definition. I wish to suggest that, while respecting Werner’s intentions, one can define the ${\text{M}}_{\mathcal{G}}$s in terms of something very much like my function BH. (It is actually thanks to my efforts to try to make clear sense of Werner’s notion of rank that I came up with the idea of defining BH for grounding trees and then to define my notion of B-rank in terms of it.)

Let $\mathcal{G}$ be a grounding graph without infinite paths. Define function W-BH(…, $\mathcal{G}$) exactly as I defined BH(…, $\mathcal{J}$), except that instead of invoking function IG(…, $\mathcal{J}$), one appeals to function W-IG(…, $\mathcal{G}$), where W-IG(G, $\mathcal{G}$) is the set of all facts H such that for some grounding tie 〈Δ, G〉 ∈  $\mathcal{G}$, H ∈ Δ. One can show that every fact in $\mathcal{G}$ belongs exactly to exactly one W-BH(α, $\mathcal{G}$) (see Proposition 2 in the “Appendix”). My suggestion is to define $\text{M}_{\mathcal{G}}\text{(F)}$ as the ordinal α such that F ∈ W-BH(α, $\mathcal{G}$).^12^

Define the *W-B-height* of a fact F in a complete grounding graph $\mathcal{G}$ for F without infinite paths as $\text{M}_{\mathcal{G}}\text{(F)}$, and define the *W-B-rank* of a fact as the smallest of its W-B-heights. On my proposed reconstruction, Werner’s definition of *being more fundamental than* goes as follows:

(W-B-MFT): F *is more fundamental*_*W-B*_* than* G iff_df_ F’s W-B-rank is smaller than G’s W-B-rank.

Say that a fact is *W-bounded* iff there are complete grounding graphs for that fact that do not have infinite paths. Just like my (B-MFT) captures a relation between bounded facts, (W-B-MFT) captures a relation between W-bounded facts.

So, (my reconstruction of) Werner’s definition and my (B-MFT) are quite similar. It can actually be established that the relations they define are *coextensive*: it can indeed be proved (with some efforts) that a fact is bounded iff it is W-bounded (see Theorem 2 in the “Appendix”) and that the B-rank of a bounded fact is identical with its W-B-rank (see Theorem 3).

The similarity between Werner’s “grounding graphs” approach and my “grounding trees” approach to the characterisation of relative fundamentality can be shown to go beyond this result. It is easy to adapt my top-down conception of relative fundamentality to Werner’s approach. Let $\mathcal{G}$ be a complete grounding graph for a fact F. Let F’s *W-T-height* in $\mathcal{G}$ be

(i) ω if there is an infinite path to F in $\mathcal{G}$;
(ii) ω if all paths to F in $\mathcal{G}$ are finite and there is no finite ordinal α such that all these paths have at most α elements;
(iii) α + 1 if there is a finite path to F in $\mathcal{G}$ with α elements such that all the other paths to F in $\mathcal{G}$ have at most α elements.

Let the *W-T-rank* of a fact F be 1 if F is not immediately grounded, and the smallest of its W-T-heights otherwise. It can be shown that for every fact that is immediately grounded, there is a complete grounding graph for that fact (see Proposition 14 in the “Appendix”). It follows that all facts have a W-T-rank. Consider then the following definition of *being more fundamental than*:

(W-T-MFT): F *is more fundamental*_*W-T*_* than* G iff_df_ F’s W-T-rank is smaller than G’s W-T-rank.

It can be shown (also with some efforts) that the T-rank of a fact is identical with its W-T-rank. Therefore, (W-T-MFT) and my (T-MFT) define coextensive relations.

These results are interesting, because they show that from an extensional point of view, it does not matter in the end whether one uses grounding trees or grounding graphs as the basic material to characterise the notion of *being more fundamental than*, be it of the top-down flavour or of the bottom-up flavour. However, I believe that my approach is to be preferred. There is indeed a good reason to dismiss the idea of using grounding graphs in the way Werner does in order to represent genealogies of facts built from connections of immediate grounding. One would expect that each grounding graph for a fact represents one particular such genealogy. This is not the case. Suppose for instance that F ∨ G is immediately grounded in F, and also in G, and that G is immediately grounded in F. Then consider the grounding graph that contains just the grounding ties 〈{F}, F ∨ G〉, 〈{G}, F ∨ G〉 and 〈{F}, G〉. This is a grounding graph for F ∨ G (which is complete if F is not immediately grounded) that represents *two* genealogies for this fact: the genealogy that goes from F straight to F ∨ G, and another one that goes from F to F ∨ G via G. Thus, some (indeed, many) grounding graphs correspond to more than one ground-theoretic genealogy, and accordingly they are not adequate tools for perspicuously representing such genealogies. Clearly, my grounding trees are better than Werner’s grounding graphs in the relevant respect: every grounding tree represents a specific genealogy (one that goes as far “down” as possible if the tree is complete).

## Being as fundamental as and being fundamental

I have defined two relations of *being more fundamental than*, one via (T-MFT) and the other one via (B-MFT). How are the sister relations of *being as fundamental as* and the sister properties of *being fundamental* to be defined?

There is little doubt that they can be defined as follows:

(T-AFA): F is *as fundamental*_*T*_* as* G iff_df_ F and G have the same T-rank.
(T-F): F is *fundamental*_*T*_ iff_df_ F is of minimal T-rank, i.e. of T-rank 1.
(B-AFA): F is *as fundamental*_*B*_* as* G iff_df_ F and G have the same B-rank.
(B-F): F is *fundamental*_*B*_ iff_df_ F is of minimal B-rank, i.e. of B-rank 1.

Relying on a suggestion by Jon Litland, Bennett (2017: p. 173) proposes to characterise *being as fundamental as* in terms of *being more fundamental than* along the following lines (unlike her, here I focus on fact-fundamentality): F is as fundamental as G just in case F and G are more fundamental than the same facts, and less fundamental than the same facts. The proposal is compelling, at least insofar as the corresponding biconditional sounds intuitively correct. Of course, (T-MFT) and (T-AFA) together entail the corresponding biconditional, and the same is true of (B-MFT) and (B-AFA) granted that we focus on bounded facts. This vindicates, if it was needed at all, the idea of adopting (T-AFA) given (T-MFT), and (B-AFA) given (B-MFT).

*Being fundamental*_*T*_ is coextensive with *being minimal for the relation of being more fundamental*_*T*_* than*. It is also coextensive with *not being immediately grounded*. If there are no transcendent facts, the property is also coextensive with *being ungrounded*. Not so if there are transcendent facts, for in this case there are facts that are fundamental_T_ but which are grounded. But this need not bother me: I offered (T-MFT) as an account that is acceptable on the assumption that there are no transcendent facts, and I offer (T-F)—and (T-AFA), for that matter—with the same qualification. These considerations about *being fundamental*_*T*_ also apply, *mutatis mutandis*, to *being fundamental*_*B*_.^13^

## Appendix

The main purpose of this appendix is to establish propositions that are mentioned in the previous sections, namely Propositions 1 and 2, Theorems 1–4, and Propositions 14 and 15. The remaining 12 propositions except one (Theorem 5) are auxiliary, they are used to establish some of the other propositions. The appendix is also of independent interest, as it establishes deep connections between two ways of modelling ground-theoretic connections that have been used by others, the tree-theoretic way advocated in this paper and also used e.g. in Correia 2014 and deRosset 2015, and the graph-theoretic way used in Werner forthcoming and also used in deRosset 2015 and several papers by Litland, starting from Litland 2015.

### On the BH functions

#### **Proposition 1**

*Let* $\mathcal{J}$ *be a grounding tree that does not generate infinite paths and let function BH be as defined in* Sect. 3. *Then for every fact F that appears in* $\mathcal{J}$, *there is a unique ordinal α such that F ∈ BH(α, *$\mathcal{J}$).

#### *Proof *

(1) *Unicity*. Suppose G is in both BH(α, $\mathcal{J}$) and BH(α*, $\mathcal{J}$) where α* ≠ α. Suppose that α* < α (the case where α < α* is exactly similar). Then either α = δ + 1 for some ordinal δ or α is a limit ordinal. Assume that α = δ + 1. Since G ∈ BH(α, $\mathcal{J}$), there must be a fact H ∈ IG(G, $\mathcal{J}$) such that H ∈ BH(δ, $\mathcal{J}$). But since G ∈ BH(α*, $\mathcal{J}$), then for all β, H ∈ BH(β, $\mathcal{J}$) only if β < α*. This is impossible since α* ≤ δ. Assume now that α is a limit ordinal. Since G ∈ BH(α*, $\mathcal{J}$), IG(G, $\mathcal{J}$) comprises only facts in BH(β, $\mathcal{J}$) for β ≤ α* < α. But since G ∈ BH(α*, $\mathcal{J}$), there is *no* α* < α such that IG(G, $\mathcal{J}$) comprises only facts in BH(β, $\mathcal{J}$) for β ≤ α*. Contradiction. (2) *Existence*. (This proof is essentially the same as the one Werner gives for the claim that his function ${\text{M}}_{\mathcal{G}}$ is defined on every fact in grounding graph $\mathcal{G}$.) Given the way BH is defined, for every fact G in $\mathcal{J}$, if every member of IG(G, $\mathcal{J}$) belongs to some BH(α, $\mathcal{J}$), then the same is true of G. So, suppose for *reductio* that for some G in $\mathcal{J}$, G belongs to no BH(α, $\mathcal{J}$). Then there is a fact G_1_ in IG(G, $\mathcal{J}$) that belongs to no BH(α, $\mathcal{J}$). But then there is a fact G_2_ in IG(G_1_, $\mathcal{J}$) that belongs to no BH(α, $\mathcal{J}$), and then a fact G_3_ in IG(G_2_, $\mathcal{J}$) that belongs to no BH(α, $\mathcal{J}$), and so on and so forth. It follows that $\mathcal{J}$ generates infinite paths—contrary to hypothesis.□

#### **Proposition 2**

* Let* $\mathcal{G}$ *be a grounding graph that does not generate infinite paths and let function W-BH be as defined in *Sect. 4*. Then for every fact F that appears in *$\mathcal{G}$ *, there is a unique ordinal α such that F ∈ W-BH(α**,* $\mathcal{G}$).

#### *Proof*

Exactly like the previous proof.□

### From (nontrivial) grounding trees to the corresponding grounding graphs

Let a *segment* of a tree be a set of nodes of the tree totally ordered by the ordering relation of the tree. Note that every segment of a tree is included in a branch of the tree, and that a branch is a maximal segment.

Remember, a grounding tie 〈Γ, F〉 is said to be *implemented* at a node x in a grounding tree iff x is a parent node that is occupied by F and the members of Γ are the facts that occupy x’s children. Say that a path is *implemented* at a segment of a grounding tree iff there is a one–one correspondence between the indices of the path and the members of the segment such that (i) every element of the path is implemented at the corresponding node of the segment and (ii) for all grounding ties 〈Γ_1_, F_1_〉 and 〈Γ_2_, F_2_〉 such that 〈Γ_1_, F_1_〉 immediately precedes 〈Γ_2_, F_2_〉 in the path, there are nodes x and y in the segment such that x is a child of y, 〈Γ_1_, F_1_〉 is implemented at x and 〈Γ_2_, F_2_〉 is implemented at y. Note that if a path is implemented at a segment of a grounding tree, then the path and the segment have the same number of elements. Also note that a path consisting of a single grounding tie is implemented at a segment consisting of a single node iff that grounding tie is implemented at that node.

Say that a path is *implemented* in a grounding tree iff it is implemented at some segment of the tree.

Let the grounding graph *corresponding* to a grounding tree / a segment of a grounding tree be the grounding graph consisting of all the grounding ties that are implemented at some nodes in the grounding tree / the segment. A grounding tree has a corresponding grounding graph iff it is nontrivial, i.e. if it has more than one node.

The following proposition is immediate:

#### **Proposition 3**

*Let* $\mathcal{J}$ *be a nontrivial grounding tree for a fact F and* $\mathcal{G}$ *its corresponding grounding graph.*

- *If a path is implemented in* $\mathcal{J}$,* then it is a path in* $\mathcal{G}$.
- $\mathcal{G}$* is a grounding graph for F.*
- $\mathcal{J}$* is complete iff* $\mathcal{G}$* is complete.*

The converse of the first point does not hold in general: there may be paths in a given grounding graph corresponding to a grounding tree that are not implemented in the tree. The converse does hold for a restricted family of grounding trees, as Proposition 4 below asserts.

Say that a grounding tree is *uniform* iff for any grounding ties 〈Γ_1_, G〉 and 〈Γ_2_, G〉 that are implemented at some nodes of the tree, Γ_1_ = Γ_2_.

#### **Proposition 4**

*Let* $\mathcal{J}$*be a nontrivial uniform grounding tree and* $\mathcal{G}$ *the corresponding grounding graph. Then every path in* $\mathcal{G}$ *is implemented in *$\mathcal{J}$.

#### *Proof *

Suppose there is a path in $\mathcal{G}$ that is *not* implemented in $\mathcal{J}$. Then there are grounding ties 〈Γ_1_, F_1_〉 and 〈Γ_2_, F_2_〉 such that 〈Γ_1_, F_1_〉 immediately precedes 〈Γ_2_, F_2_〉 in the path, so that F_1_ ∈ Γ_2_, and such that (P) for all nodes x and y in $\mathcal{J}$ such that x is a child of y, either 〈Γ_1_, F_1_〉 is not implemented at x or 〈Γ_2_, F_2_〉 is not implemented at y. Let y be a node in $\mathcal{J}$ at which 〈Γ_2_, F_2_〉 is implemented. Since F_1_ ∈ Γ_2_, F_1_ occupies a child x of y. Now since 〈Γ_1_, F_1_〉 is implemented at some node in $\mathcal{J}$ and $\mathcal{J}$ is uniform, 〈Γ_1_, F_1_〉 must be implemented at x. But this is impossible given (P).□

#### **Proposition 5**

*Let* $\mathcal{J}$ *be a nontrivial uniform grounding tree for a fact F and* $\mathcal{G}$ *the corresponding grounding graph. Then F’s T-height in* $\mathcal{J}$ *is identical with F’s W-T-height in* $\mathcal{G}$.

#### *Proof*

Let t be F’s T-height in $\mathcal{J}$ and let g be F’s W-T-height in $\mathcal{G}$. Case 1: there is no path $\mathcal{P}$ in $\mathcal{G}$ such that (i) $\mathcal{P}$ has a finite number α of elements and (ii) all the other paths have a number of elements that is smaller than or equal to α. In this case, g = ω. By Proposition 4, there is no branch ${\mathcal{B}}$ in $\mathcal{J}$ such that (i) ${\mathcal{B}}$ has a finite number α of elements and (ii) all the others paths have a number of elements that is smaller than or equal to α. It follows that t = ω. Case 2: there is a path $\mathcal{P}$ in $\mathcal{G}$ such that (i) $\mathcal{P}$ has a finite number α of elements and (ii) all the other paths have a number of elements that is smaller than or equal to α. In this case, g = α + 1. By Proposition 4, t ≥ α + 1. We cannot have t > α + 1, for otherwise by Proposition 3 (first point) $\mathcal{G}$ would contain a graph with more than α elements. Therefore, t = α + 1.□

#### **Proposition 6**

*Let* $\mathcal{J}$ *be a nontrivial grounding tree for a fact F that does not generate infinite paths and* $\mathcal{G}$ *the corresponding grounding graph. Then F’s B-height in* $\mathcal{J}$ *is identical with F’s W-B-height in* $\mathcal{G}$.

#### *Proof*

The condition on $\mathcal{J}$ implies that F has a B-height in $\mathcal{J}$, and given that $\mathcal{J}$ does not generate infinite paths, no path in $\mathcal{G}$ is infinite and therefore F has a W-B-height in $\mathcal{G}$. To get the result it suffices to prove that for any nonnull ordinal α, BH(α, $\mathcal{J}$) = W-BH(α, $\mathcal{G}$). But this is immediate since the facts in $\mathcal{J}$ and the facts in $\mathcal{G}$ are the same, and for any of these facts G, IG(G, $\mathcal{J}$) = W-IG(G, $\mathcal{G}$).□

### From uniform grounding graphs to grounding trees they correspond to

Say that a grounding graph is *uniform* iff for any grounding ties 〈Γ_1_, G〉 and 〈Γ_2_, G〉 in the graph, Γ_1_ = Γ_2_. Clearly:

#### **Proposition 7**

*If* $\mathcal{G}$ *is the grounding graph that corresponds to grounding tree* $\mathcal{J}$, *then* $\mathcal{G}$ *is uniform iff* $\mathcal{J}$ *is uniform*.

#### **Proposition 8**

*Let* $\mathcal{G}$ *be a uniform grounding graph for a fact F. Then there is a grounding tree* $\mathcal{J}$ *for F to which* $\mathcal{G}$ *corresponds*.

#### *Proof*

I build a grounding tree for F such that a grounding tie 〈Γ, G〉 is implemented at some node of the tree iff 〈Γ, G〉 ∈ $\mathcal{G}$. Where G is a fact that appears as the head of a grounding tie in $\mathcal{G}$, I let Δ(G) be the unique set of facts Γ such that 〈Γ, G〉 ∈ $\mathcal{G}$ (since the graph is uniform, the definite description is proper).

If the set of all the facts that appear in $\mathcal{G}$ is finite, let κ be ℵ_0_, and if it is infinite, let κ be its cardinality. I suppose given a set S of objects, distinct from the facts that appear in $\mathcal{G}$, whose cardinality card(S) is the smallest infinite cardinal number that is larger than κ (so if κ is ℵ_0_, then card(S) = ℵ_1_, if κ is ℵ_1_, then card(S) = ℵ_2_, and so on). The tree to be constructed will have its nodes taken from S, and the cardinality hypothesis is meant to ensure that there are enough nodes to perform the construction.

Let a *basic tree* be a tree whose nodes are in S and whose branches are all of cardinality 2, and let a *basic grounding tree* be a grounding tree with the following features: (i) its underlying tree is basic, (ii) there is a grounding tie 〈Δ(G), G〉 in $\mathcal{G}$ that is implemented at the node of the tree such that no fact in Δ(G) occupies more than one its leaves. The grounding tie 〈Δ(G), G〉 which satisfies condition (ii) is called *the grounding tie of the basic grounding tree*. Clearly, the number of nodes in a basic grounding tree is smaller than or equal to κ.

Let us represent labelled trees as triples 〈T, < , lab〉, where 〈T, <〉 is a tree and lab a function that assigns a fact to each member of T. I build a series of grounding trees indexed by the nonnull finite ordinals $\mathcal{J}$_1_ = 〈T_1_, < _1_, lab_1_〉, $\mathcal{J}$_2_ = 〈T_2_, < _2_, lab_2_〉, … as follows:

Let $\mathcal{J}$_1_ be a basic grounding tree whose grounding tie is 〈Δ(F), F〉. Note that the number of nodes in $\mathcal{J}$_1_ is smaller than or equal to κ (see previous remark). Assume that $\mathcal{J}$_n_ has been constructed and that the number of nodes in $\mathcal{J}$_n_ is smaller than or equal to κ. If $\mathcal{J}$_n_’s leaves are all occupied by facts that are not heads of grounding ties in $\mathcal{G}$, then I let $\mathcal{J}$_n+1_ be $\mathcal{J}$_n_. Otherwise, I construct $\mathcal{J}$_n+1_ as an extension of $\mathcal{J}$_n_ along the following lines. Let a *target* be a pair 〈x, G〉 such that x is a leaf in $\mathcal{J}$_n_, G occupies x and 〈Δ(G), G〉 ∈ $\mathcal{G}$. I associate to each target 〈x, G〉 a basic grounding tree $\mathcal{J}$(x, G) with root x whose grounding tie is 〈Δ(G), G〉, in such a way that the leaves of any $\mathcal{J}$(x, G) are (i) distinct from the nodes in $\mathcal{J}$_n_ and (ii) distinct from the leaves in any $\mathcal{J}$(y, H) where y ≠ x. Here the hypothesis that κ is smaller than card(S) is crucial, since it ensures that there are enough objects in S to provide the relevant leaves (since the number of nodes in $\mathcal{J}$_n_ is smaller than or equal to κ and the number of nodes in a grounding tree whose grounding tie is in $\mathcal{G}$ is smaller than or equal to κ, the number of required leaves is smaller than or equal to κ × κ = κ). Call these basic grounding trees the *new trees*. $\mathcal{J}$_n+1_ is the result of appending all the new trees to $\mathcal{J}$_n_. More formally, the components of $\mathcal{J}$_n+1_ are defined as follows:

- T_n+1_ = T_n_ ∪ {x: x is a leaf in a new tree}.
- x < _n+1_ y iffeither x, y ∈ T_n_ and x < _n_ y,
  or y is a leaf in a new tree and x = y’s parent in that tree,
  or y is a leaf in a new tree and x < _n_ y’s parent in that tree.
- Lab_n+1_(x) = Lab_n_(x) if x is not a leaf in a new tree;
  Lab_n+1_(x) = the fact that occupies x in the corresponding new tree otherwise.

Note that the number of nodes in $\mathcal{J}$_n+1_ is smaller than or equal to κ. Clearly, for every nonnull finite ordinal, T_n_ ⊆ T_n+1_, < _n_ ⊆  < _n+1_ and lab_n_ ⊆ lab_n+1_, and every $\mathcal{J}$_n_ is a grounding tree for F.

Let T be the union of the T_n_s, < the union of the < _n_s, and lab the union of the lab_n_s. Let $\mathcal{J}$ be 〈T, < , lab〉. Given that every $\mathcal{J}$_n_ is a grounding tree for F, $\mathcal{J}$ is also a grounding tree for F.

By construction, if a grounding tie is implemented at some node of $\mathcal{J}$, then this grounding tie is in $\mathcal{G}$. The converse is also true. For consider a grounding tie 〈Δ(G), G〉 ∈ $\mathcal{G}$. Then there must be a path to F in $\mathcal{G}$ whose first element is 〈Δ(G), G〉. Any such path must have a finite number of elements. If there is a 1-element path from 〈Δ(G), G〉 to F in $\mathcal{G}$, then 〈Δ(G), G〉 = 〈Δ(F), F〉 and we know that this grounding tie is implemented at the root of $\mathcal{J}$. Let us show by induction that if there is an n-element path from 〈Δ(G), G〉 to F in $\mathcal{G}$, with n ≥ 2, then 〈Δ(G), G〉 is implemented at some leaf in $\mathcal{J}$_n−1_. (i) Suppose that there is a 2-element path from 〈Δ(G), G〉 to F in $\mathcal{G}$. This means that G ∈ Δ(F), and hence that 〈Δ(G), G〉 is implemented at some leaf in $\mathcal{J}$_1_. (ii) Suppose the proposition holds for n and assume that there is an (n + 1)-element path from 〈Δ(G), G〉 to F in $\mathcal{G}$. Then there is a grounding tie 〈Δ(H), H〉 in $\mathcal{G}$ such that (a) G ∈ Δ(H) and (b) there is an n-element path from 〈Δ(H), H〉 to F in $\mathcal{G}$. By induction hypothesis and (b), 〈Δ(H), H〉 is implemented at some leaf in $\mathcal{J}$_n−1_. But then by (a), 〈Δ(G), G〉 is implemented at some leaf in $\mathcal{J}$_n_.□

### From complete to uniform complete

#### **Proposition 9**

*If there is a complete grounding graph for a fact F such that F’s W-T-height in that graph is g, then there is a uniform complete grounding graph for F such that F’s W-T-height in that graph is smaller than or equal to g.*

#### *Proof *

Let $\mathcal{G}$ be a non-uniform complete grounding graph for F such that F’s W-T-height in $\mathcal{G}$ is g. Let a *problematic* fact be a fact G such that $\mathcal{G}$ contains two distinct grounding ties 〈Γ_1_, G〉 and 〈Γ_2_, G〉, and associate to each problematic fact G a given set of facts Δ(G) such that 〈Δ(G), G〉 ∈ $\mathcal{G}$. Let $\mathcal{G}$* be the grounding graph whose members are the members of $\mathcal{G}$* without the ties 〈Γ, G〉 where G is problematic and Γ ≠ Δ(G). $\mathcal{G}$* is clearly uniform and complete. But it need not be a graph for F: some tie in $\mathcal{G}$* may fail to belong to a path to F. Consider the set of all the paths for F in $\mathcal{G}$* and let $\mathcal{G}$** be the grounding graph whose members are all the grounding ties that are elements of these paths. $\mathcal{G}$** is nonempty since it contains 〈Δ(F), F〉. By construction it is of course a grounding graph for F and it is uniform. Remains to show that it is complete and that F’s W-T-height in $\mathcal{G}$** is smaller than or equal to g. The latter point is obvious since by construction every path to F in $\mathcal{G}$** is a path to F in $\mathcal{G}$. For completeness, let G be a fact that is immediately grounded and that belongs to the tail of some grounding tie 〈Γ, H〉 ∈ $\mathcal{G}$**. Then 〈Γ, H〉 belongs to some path to F in $\mathcal{G}$*. Since $\mathcal{G}$ is complete, there is a grounding tie whose head is G in $\mathcal{G}$*. Since 〈Γ, H〉 belongs to some path to F in $\mathcal{G}$*, such grounding ties also belong to some path to F in $\mathcal{G}$*, and therefore they belong to $\mathcal{G}$**. Hence, $\mathcal{G}$** is complete.□

#### **Proposition 10**

*If there is a complete grounding graph without infinite paths for a fact F such that F’s W-B-height in that graph is g, then there is a uniform complete grounding graph without infinite paths for F such that F’s W-B-height in that graph is smaller than or equal to g.*

#### *Proof*

Let $\mathcal{G}$ be a complete grounding graph without infinite paths for F such that F’s W-B-height in that graph is g and let $\mathcal{G}$** be defined in terms of $\mathcal{G}$ as in the previous proof. Then $\mathcal{G}$** ⊆ $\mathcal{G}$. It follows that $\mathcal{G}$** does not have infinite paths and hence that F has a W-B-height in $\mathcal{G}$**. I establish by induction the following proposition, which clearly entails that F’s W-B-height in $\mathcal{G}$** is smaller than or equal to g:

For all ordinals α and facts G featuring in $\mathcal{G}$** such that G ∈ W-BH(α, $\mathcal{G}$), there is an ordinal δ ≤ α such that G ∈ W-BH(δ, $\mathcal{G}$**). Note that since $\mathcal{G}$** ⊆ $\mathcal{G}$, W-IG(G, $\mathcal{G}$**) ⊆ W-IG(G, $\mathcal{G}$). For α = 1, the proposition directly follows from this latter fact. Suppose the proposition holds for all β ≤ α and assume that G features in $\mathcal{G}$** and G ∈ W-BH(α + 1, $\mathcal{G}$). Then W-IG(G, $\mathcal{G}$) ⊆  ∪ _β ≤ α_ W-BH(β, $\mathcal{G}$), and hence W-IG(G, $\mathcal{G}$**) ⊆  ∪ _β ≤ α_ W-BH(β, $\mathcal{G}$). By induction hypothesis we get that W-IG(G, $\mathcal{G}$**) ⊆  ∪ _β ≤ α_ W-BH(β, $\mathcal{G}$**). It follows that G ∈ W-BH(δ, $\mathcal{G}$**) for some δ ≤ α + 1. Suppose finally that the proposition holds for all β < α where α is a limit ordinal, and assume that G features in $\mathcal{G}$** and G ∈ W-BH(α, $\mathcal{G}$). Then W-IG(G, $\mathcal{G}$) ⊆  ∪ _β < α_ W-BH(β, $\mathcal{G}$). Reasoning as in the previous case yields the conclusion that G ∈ W-BH(δ, $\mathcal{G}$**) for some δ ≤ α.□

A *subtree* of a tree 〈T, <〉 is a pair 〈U, <〉 such that for some node x in T, U = {x} ∪ {y ∈ T: x < y}. Subtrees of a tree are themselves trees, and subtrees of a bush are bushes.

A *labelled subtree* of a labelled tree $\mathcal{J}$ is a labelled tree whose underlying tree is a subtree of $\mathcal{J}$’s underlying tree, such that a fact occupies a node in iff it occupies that node in $\mathcal{J}$. Labelled subtrees of a grounding tree are themselves grounding trees.

Where α is an ordinal number, the *α-level* of a bush is the set of all nodes of the tree that have α predecessors.

#### **Proposition 11**

*If there is a complete grounding tree for a fact F of finite T-height t, then there is a uniform complete grounding tree for F of T-height smaller than or equal to t.*

#### *Proof*

Let $\mathcal{J}$ be a grounding tree for a fact F of finite T-height t and suppose it is not uniform. Note that since $\mathcal{J}$ is not uniform, t > 2. A fact G is said to be *problematic* iff some distinct grounding ties 〈$\Gamma_1$, G〉 and 〈$\Gamma_2$, G〉 are implemented at some nodes in $\mathcal{J}$. The idea is now to build a new grounding tree $\mathcal{J}$* for F from $\mathcal{J}$ with the desired properties by “eliminating” problematic facts step by step. I illustrate the construction with t = 4—how to deal with the general case will then be clear enough.

$\mathcal{J}$’s 0-level contains only $\mathcal{J}$’s root, $\mathcal{J}$’s 3-level only contains nodes that are among $\mathcal{J}$’s leaves, and $\mathcal{J}$’s α-levels for α > 3 are all empty. Only $\mathcal{J}$’s 0-level, 1-level and 2-level contain parent nodes, and therefore each problematic fact must occupy nodes that are in some of these levels. I accordingly build $\mathcal{J}$* in three steps, first building a tree $\mathcal{J}$+ from $\mathcal{J}$, then a tree $\mathcal{J}$++ from $\mathcal{J}$+ , and finally $\mathcal{J}$* from $\mathcal{J}$++:

1. If no problematic fact occupies more than one node in $\mathcal{J}$’s 2-level, let $\mathcal{J}$+ be $\mathcal{J}$. Otherwise, do the following with each problematic fact G of the sort in question. Consider all the labelled subtrees of $\mathcal{J}$ whose roots are in $\mathcal{J}$’s 2-level and are occupied by G. Since $\mathcal{J}$ is complete, none of these labelled subtrees is of T-height 1, and therefore they are all of T-height 2. Arbitrarily pick out one of them and replace every other labelled subtree whose root is in $\mathcal{J}$’s 2-level and is occupied by G by this chosen subtree. (I am a bit sloppy here: the replacements should be done using labelled subtrees *isomorphic* to the chosen subtree, such that no two of these subtrees share a node and none of these subtrees shares a node (except perhaps its root) with $\mathcal{J}$. Here, and indeed also in the rest of the proof below, I am less careful than I was in the proof of Proposition 8.) $\mathcal{J}$+ is the resulting tree. Note that $\mathcal{J}$+ is a grounding tree for F, that its T-height is 4, that no problematic fact occupies more than one node in  $\mathcal{J}$+’s 2-level, and finally that $\mathcal{J}$+ is complete.
2. If no problematic fact occupies more than one node in  $\mathcal{J}$+’s 1-level or 2-level, let $\mathcal{J}$++ be $\mathcal{J}$+ . Otherwise, do the following with each problematic fact G of the sort in question. Consider all the labelled subtrees of  + whose roots are in $\mathcal{J}$+’s 1-level or 2-level and are occupied by G. Since $\mathcal{J}$ is complete, none of these labelled subtrees is of T-height 1, and therefore they are all of T-height 2 or 3. There are three possible cases:
  (i) One of these labelled subtrees was used in some replacement at the previous step.
  (ii) (i) is not the case and one of these labelled trees is of T-height 2.
  (iii) (i) is not the case and none of these labelled trees is of T-height 2.
  I define grounding tree $\mathcal{J}$(G) as follows: in case (i), $\mathcal{J}$(G) is the labelled subtree that was used in some replacement; in case (ii), $\mathcal{J}$(G) is an arbitrary labelled subtree of T-height 2 among those in question; in case (iii), $\mathcal{J}$(G) is an arbitrary labelled subtree of T-height 3 among those in question. In cases (i) and (ii), thus, $\mathcal{J}$(G)’s T-height is 2, whereas in case (iii), it is 3. Replace every labelled subtree of $\mathcal{J}$+ distinct from $\mathcal{J}$(G) whose root is in $\mathcal{J}$+’s 1-level or 2-level and is occupied by G by $\mathcal{J}$(G). $\mathcal{J}$++ is the resulting tree. Note that  $\mathcal{J}$++ is a grounding tree for F, that its T-height is either 4 or 3, that no problematic fact occupies more than one node in $\mathcal{J}$++’s 1-level or 2-level, and finally that $\mathcal{J}$++ is complete.
3. If F does not occupy a node in  $\mathcal{J}$++ other than $\mathcal{J}$++’s root, then let $\mathcal{J}$* be $\mathcal{J}$++ . Otherwise, let x be a node in  $\mathcal{J}$++ distinct from $\mathcal{J}$++’s root that is occupied by F and let $\mathcal{J}$* be the labelled subtree of $\mathcal{J}$++ whose root is x. In either case, $\mathcal{J}$* is a grounding tree for F, its T-height is smaller than or equal to 4, it is uniform, and it is complete.□

#### **Proposition 12**

*If there is a complete grounding tree that does not generate infinite paths and that is of B-height t, then there is a uniform complete grounding tree for the same fact that does not generate infinite paths and that is of B-height smaller than or equal to t.*

#### *Proof*

Let $\mathcal{J}$ be a grounding tree for a fact F that satisfies the antecedent. If $\mathcal{J}$ is trivial, i.e. has only one node, then it satisfies the consequent. Suppose $\mathcal{J}$ is nontrivial and let $\mathcal{G}$ be the corresponding grounding graph. Since $\mathcal{J}$ does not generate infinite paths, $\mathcal{G}$ does not have infinite paths and accordingly F has a W-B-height in $\mathcal{G}$. By Propositions 3 and 6, $\mathcal{G}$ is a complete grounding graph for F and F’s W-B-height in $\mathcal{G}$ is t. By Proposition 10, there is a uniform complete grounding graph $\mathcal{G}$* for F such that F’s W-B-height t* in $\mathcal{G}$* is smaller than or equal to t. Thanks to Proposition 8, we know that there is a grounding tree $\mathcal{J}$* for F to which $\mathcal{G}$* corresponds. Since $\mathcal{G}$* has no infinite paths, $\mathcal{J}$* does not generate infinite paths. By Propositions 3, 6 and 7, $\mathcal{J}$* is uniform, complete and its B-height is t* and hence is smaller than or equal to t.□

### Comparing ranks

#### **Theorem 1**

*The T-rank of a fact is identical with its W-T-rank.*

#### *Proof*

Let F be an arbitrary fact. F’s T-rank is 1 iff F’s W-T-rank is 1. To get the general result it suffices to establish the following two points:

1. If there is a nontrivial complete grounding tree for F of T-height t, then there is a complete grounding graph for F such that F’s W-T-height in that graph is smaller than or equal to t.
2. If there is a complete grounding graph for F such that F’s W-T-height in that graph is g, then there is a complete grounding tree for F of T-height smaller than or equal to g.

For the first point, suppose the antecedent is true and let $\mathcal{J}$ be a witness. Suppose t = ω. Consider the grounding graph $\mathcal{G}$ corresponding to $\mathcal{J}$. By Proposition 3, $\mathcal{G}$ is a complete grounding graph for F, and F’s W-T-height $\mathcal{G}$ —like the W-T-height of any fact—is smaller than or equal to ω. Suppose now t is finite. By Proposition 11, there is a uniform complete grounding tree $\mathcal{J}$* for F of T-height t* ≤ t. Since F’s T-rank is not 1, then $\mathcal{J}$* is nontrivial. Let $\mathcal{G}$ be the grounding graph corresponding to $\mathcal{J}$*. By Proposition 3, $\mathcal{G}$ is complete, and by Proposition 5, F’s W-T-height in $\mathcal{G}$ is t* and hence is smaller than or equal to t.

For the second point, suppose the antecedent is true and let $\mathcal{G}$ be a witness. By Proposition 9, there is a uniform complete grounding graph $\mathcal{G}$* for F such that F’s W-T-height g* is smaller than or equal to g. By Propositions 8, 7, 5 and 3, there is a complete grounding tree $\mathcal{J}$ for F whose T-height is g* and hence is smaller than or equal to g.□

#### **Theorem 2**

*A fact is bounded iff it is W-bounded.*

#### *Proof*

Suppose F is bounded, i.e. that there is a complete grounding tree $\mathcal{J}$ for F that does not generate infinite paths. Let $\mathcal{G}$ be the corresponding grounding graph. By Proposition 3, it is a complete grounding graph for F, and it does not have infinite paths since $\mathcal{J}$ does not generate such paths. Hence, F is W-bounded. Conversely, suppose F is W-bounded and let $\mathcal{G}$ be a complete grounding graph for F without infinite paths. By Proposition 10, there is a uniform complete grounding graph $\mathcal{G}$ * for F without infinite paths. By Proposition 8, there is a grounding tree $\mathcal{J}$ for F to which $\mathcal{G}$* corresponds. By Proposition 3, $\mathcal{J}$ is complete, and it does not generate infinite paths since $\mathcal{G}$* does not have such paths.□

#### **Theorem 3**

*The B-rank of a bounded fact is identical with its W-B-rank.*

#### *Proof*

Let F be an arbitrary bounded fact. F’s B-rank is 1 iff F’s W-B-rank is 1. To get the general result it suffices to establish the following two points:

1. If there is a nontrivial complete grounding tree for F that does not generate infinite paths of B-height t, then there is a complete grounding graph for F without infinite paths such that F’s W-B-height in that graph is smaller than or equal to t.
2. If there is a complete grounding graph for F without infinite paths such that F’s W-B-height in that graph is g, then there is a complete grounding tree for F that does not generate infinite paths of B-height smaller than or equal to g.

For the first point, suppose the antecedent is true and let $\mathcal{J}$ be a witness. By Proposition 12 there if a uniform complete grounding tree $\mathcal{J}$* for F that does not generate infinite paths of B-height t* ≤ t. Since F’s B-rank is not 1, then $\mathcal{J}$* is nontrivial. Let $\mathcal{G}$ be the grounding graph corresponding to $\mathcal{J}$*. Since $\mathcal{J}$ does not generate infinite paths, $\mathcal{G}$ does not have such paths. By Proposition 3, $\mathcal{G}$ is complete, and by Proposition 6, F’s W-B-height in $\mathcal{G}$ is t* and hence is smaller than or equal to t.

For the second point, suppose the antecedent is true and let $\mathcal{G}$ be a witness. By Proposition 10, there is a uniform complete grounding graph $\mathcal{G}$* for F without infinite paths of such that F’s W-B-height g* is smaller than or equal to g. By Propositions 8, 6 and 3, there is a complete grounding tree $\mathcal{J}$ for F that does not generate infinite paths whose B-height is g* and hence is smaller than or equal to g. □

#### **Proposition 13**

*Let α be a nonnull finite ordinal. Then for all uniform complete grounding trees* $\mathcal{J}$*,* $\mathcal{J}$ *has T-height α iff* $\mathcal{J}$ *has B-height α*.

#### *Proof*

By induction on α. The proposition is immediate if α = 1. Suppose the proposition holds up to α ≥ 1 and let $\mathcal{J}$ be a uniform complete grounding tree for a fact F. Let J be the set of nodes that are children of $\mathcal{J}$’s root, and for all j ∈ J, let $\mathcal{J}$_j_ be the labelled subtree of $\mathcal{J}$ whose root is j and G_j_ the fact that occupies j. Note that since $\mathcal{J}$ is uniform and complete, so are the $\mathcal{J}$_j_s. Also note that if $\mathcal{J}$ has a finite T-height, then it does not generate infinite paths (see Proposition 4) and hence it has a B-height.

Suppose first that $\mathcal{J}$ has T-height α + 1. For all j ∈ J, let α_j_ be $\mathcal{J}$_j_’s T-height. Then for all j ∈ J, α_j_ ≤ α, and for some j ∈ J, α_j_ = α. By induction hypothesis, then, α_j_ is $\mathcal{J}$_j_’s B-height for all j ∈ J. That is to say, G_j_ ∈ BH(α_j_, $\mathcal{J}$_j_) for all j ∈ J. Given that $\mathcal{J}$ is uniform and complete, one can easily prove by induction on β the following proposition:

(P) For every nonnull finite ordinal β, every j ∈ J and every G that appears in $\mathcal{J}$_j_, G ∈ BH(β, $\mathcal{J}$_j_) iff G ∈ BH(β, $\mathcal{J}$).

Given (P), G_j_ ∈ BH(α_j_, $\mathcal{J}$) for all j ∈ J. Because $\mathcal{J}$ is uniform, IG(F, $\mathcal{J}$) = {G_j_: j ∈ J}, and so we have that F ∈ BH(α + 1, $\mathcal{J}$), and hence that $\mathcal{J}$ has B-height α + 1.

Conversely, suppose that $\mathcal{J}$ has B-height α + 1, i.e. that F ∈ BH(α + 1, $\mathcal{J}$). For all j ∈ J, let α_j_ be $\mathcal{J}$_j_’s B-height. Then G_j_ ∈ BH(α_j_, $\mathcal{J}$_j_) for all j ∈ J. Given (P), it follows that G_j_ ∈ BH(α_j_, $\mathcal{J}$) for all j ∈ J. Because $\mathcal{J}$ is uniform, IG(F, $\mathcal{J}$) = {G_j_: j ∈ J}, and so we have that for all j ∈ J, α_j_ ≤ α, and for some j ∈ J, α_j_ = α. By induction hypothesis, then, α_j_ is $\mathcal{J}$_j_’s T-height for all j ∈ J. It follows that $\mathcal{J}$ has T-height α + 1.□

#### **Theorem 4**

*Let α be a nonnull finite ordinal and F a fact. F has T-rank α iff F has B-rank α.*

#### *Proof*

By induction on α. The proposition is immediate if α = 1. Suppose the proposition holds up to α ≥ 1 and suppose F has T-rank α + 1. This means that there is a complete grounding tree $\mathcal{J}$ for F of T-height α + 1 and that no other complete grounding tree for F has a smaller T-height. Given Proposition 11, one can assume that $\mathcal{J}$ is uniform. By Proposition 13, $\mathcal{J}$ has B-height α + 1. Now by Propositions 12 and 13, there cannot be a complete grounding tree for F of B-height smaller than α + 1. Hence, F has B-rank α + 1. Conversely, Suppose F has B-rank α + 1. This means that there is a complete grounding tree $\mathcal{J}$ for F of B-height α + 1 and that no other complete grounding tree for F has a smaller B-height. Given Proposition 12, one can assume that $\mathcal{J}$ is uniform. By Proposition 13, $\mathcal{J}$ has T-height α + 1. Now by Propositions 11 and 13, there cannot be a complete grounding tree for F of T-height smaller than α + 1. Hence, F has T-rank α + 1.□

#### **Theorem 5**

*Let α be a nonnull finite ordinal and F a fact. F has W-T-rank α iff F has W-B-rank α.*

#### *Proof*

From Theorems 1, 3 and 4.□

### From being immediately grounded to having a complete uniform grounding graph / tree

#### **Proposition 14**

*If a fact is immediately grounded, then there is a complete uniform grounding graph for that fact*.

#### *Proof*

Suppose F is immediately grounded in Γ. I build a series of graphs indexed by the nonnull finite ordinals $\mathcal{G}$_1_, $\mathcal{G}$_2_, … as follows:

- $\mathcal{G}$_1_ is the set whose sole member is the grounding tie 〈Γ, F〉.
- If $\mathcal{G}$_n_ is complete, then $\mathcal{G}$_n+1_ =  $\mathcal{G}$_n_. Otherwise, associate to each fact G in b($\mathcal{G}$_n_) that is immediately grounded a grounding tie 〈Δ, G〉, and let $\mathcal{G}$_n+1_ be the result of adding all these grounding ties to $\mathcal{G}$_n+1_.

By construction, thus, $\mathcal{G}$_n_ ⊆ $\mathcal{G}$_n+1_ for every nonnull finite ordinal n. Let $\mathcal{G}$ be the union of all these graphs. $\mathcal{G}$ is a grounding graph. I want to show that (a) it is a grounding graph for F, (b) it is uniform and (c) it is complete.

For (a), I first prove by induction that every $\mathcal{G}$_n_ is a grounding graph for F. This is of course the case for $\mathcal{G}$_1_. Suppose this is the case for $\mathcal{G}$_n_, and take a grounding tie 〈Δ, G〉 in $\mathcal{G}$_n+1_. If 〈Δ, G〉 ∈ $\mathcal{G}$_n_, then by induction hypothesis 〈Δ, G〉 is an element of a path to F in $\mathcal{G}$_n+1_. Suppose 〈Δ, G〉 ∉ $\mathcal{G}$_n_. Then there is a grounding tie 〈Δ′, G′〉 in $\mathcal{G}$_n_ such that G ∈ Δ′. By induction hypothesis, 〈Δ′, G′〉 is an element of a path to F in $\mathcal{G}$_n_. Take one such path, remove all the elements that precede a given occurrence of 〈Δ′, G′〉 in the path, and put 〈Δ, G〉 right before this occurrence. The result is a path to F in $\mathcal{G}$_n+1_ of which 〈Δ, G〉 is an element. Hence, $\mathcal{G}$_n+1_ is a grounding graph for F. Given this, it is clear that $\mathcal{G}$ is a grounding graph for F.

For (b), things are clear since if a grounding tie 〈Δ, G〉 has been introduced in the construction at step n, no distinct grounding tie of type 〈Δ′, G〉 can be introduced at a later step n + k, since then G already appears as the head of a grounding tie in n + k − 1.

For (c), suppose for *reductio* that $\mathcal{G}$ is *not* complete. This means that there is a fact G in $\mathcal{G}$ that (i) is not a head and (ii) is immediately grounded. Let 〈Δ, H〉 be a grounding tie in $\mathcal{G}$ such that G ∈ Δ. Then 〈Δ, H〉 is in some $\mathcal{G}$_n_. But then by construction, $\mathcal{G}$_n+1_ contains a grounding tie 〈Λ, G〉, and therefore G is after all a head in $\mathcal{G}$. Contradiction.□

#### **Proposition 15**

*If a fact is immediately grounded, then there is a complete uniform grounding tree for that fact.*

#### *Proof*

From Propositions 14, 8, 7 and 3. □

## Footnotes

1 In this paper I focus on fact-fundamentality and on grounding understood as relating facts. I will be neutral on whether in this context facts should be taken to be true propositions or obtaining states of affairs. I will assume that grounding is factive, i.e. that grounding can only be exemplified by *true* propositions or *obtaining* states of affairs. This is compatible with the view that factive grounding should be understood in terms of a more basic non-factive notion of grounding (see Fine 2012 on this point and more generally on the factive vs non-factive distinction). I believe that my accounts of fundamentality in terms of grounding can also be formulated, *mutatis mutandis*, if both fundamentality and grounding are taken to be expressed by means of sentential operators instead of predicates (see Correia 2010 on the operator vs predicate approaches to grounding).

2 Fact-fundamentality need not be understood in terms of grounding. Sider 2011 defines a fundamental fact as a truth that carves reality at its joints, and Correia forthcoming explores the view that it is grounding that should be understood in terms of a previously given notion of fact-fundamentality. Both views strike me as viable, they simply focus on notions of fundamentality that are distinct from the one I am interested in here. (In Correia forthcoming, which I wrote long before starting to think seriously about the tree-theoretic characterisations that I offer here, I explained that I could not think of any plausible way of characterising relative fundamentality in terms of grounding, but I did not exclude that some such characterisations could be found.) I should stress here that I also believe that fundamentality does not boil down to fact-fundamentality. I take it that Sider’s (2011) notion of fundamentality has a legitimate range of application that goes far beyond facts, and that the same is true of some notions of fundamentality linked to ontological dependence rather than grounding (see Correia 2008).

3 Bennett is neutral on whether grounding relates fact-like entities only or entities of any ontological categories. As I stressed in footnote 1, I am not.

4 Dasgupta 2014 and Litland 2016 both defend the non-orthodox view that grounding can also be many-many. I leave this idea aside in this paper.

5 This example is similar to one given in Dixon 2016: pp. 449–450 to illustrate a different point. The dense chain of grounds involving determinables / determinates mentioned in Correia forthcoming: Sect. 2.2 provides a further example.

6 See e.g. Jech 2002: p. 114. I slightly depart from Jech’s presentation, and I employ standard terminology of graph theory.

7 See Bennett 2017: p. 40 for a formulation of the principle generalised to arbitrary building relations. See also Bennett 2011: p. 1, Correia forthcoming: Sect. 1, Griffith 2018: p. 394, Koslicki 2015: p. 306, Moran 2018: p. 361, Rabin 2018: p. 42, Raven 2012: p. 689, Rosen 2010: p. 116 and Wilson 2012: p. 1. It is because Bennett endorses the generalised principle that she included clause (2) in the *definiens* of her definition of *being more fundamental than* (see Sect. 1).

8 I borrow the term ‘path’ and the notion it expresses to Werner forthcoming.

9 Generating an infinite path is not the same thing as having an infinite branch. If a grounding tree has an infinite branch, then it generates an infinite path, but the converse may fail: consider a simple grounding tree of T-height 2 for a fact F such that F occupies not only the root of the tree but also one of its leaves.

10 See e.g. Cameron 2008: pp. 1–2 and Dixon 2016: pp. 251–255. Whereas standard presentations of the ground-theoretic versions of the instantiation regress do not specify that the links of ground between adjacent items are immediate, it is important for my purposes that the links be of that sort.

11 Werner supplements his definition of *being more fundamental than* with the claim that grounding can be characterised in terms of immediate grounding along the following lines: F is grounded in the members of just in case there is a grounding graph $\mathcal{G}$ for F, without infinite paths to F, such that Γ = b($\mathcal{G}$). However, this characterisation is not essential to Werner’s definition of *being more fundamental than*. Note that if the characterisation is correct, then there cannot be transcendent facts. This point could be used to argue against it.

12 To be completely accurate, the minimal value of Werner’s ${\text{M}}_{\mathcal{G}}$ is 0 while on my definition it is 1, but the difference is immaterial for the purpose of defining *being more fundamental than*. I chose to define the function the way I did in order to make comparisons with my framework more straightforward.

13 I am grateful to the audience of an *eidos* meeting for useful comments on an ancestor of this paper and to Maria Scarpati for detailed comments on another version. A reviewer for this journal helped me improve the paper to a considerable extent. Work on the paper was supported by the Swiss National Science Foundation (project BSCGI0_157792).
